# Comparison of cytotoxicity effects induced by four different types of nanoparticles in human corneal and conjunctival epithelial cells

**DOI:** 10.1038/s41598-021-04199-3

**Published:** 2022-01-07

**Authors:** Xiangzhe Li, Boram Kang, Youngsub Eom, Jingxiang Zhong, Hyung Keun Lee, Hyo Myung Kim, Jong Suk Song

**Affiliations:** 1 Department of Ophthalmology, Guro Hospital, Korea University College of Medicine, 80, Guro-dong, Guro-gu, Seoul, 152–703 South Korea; 2 Department of Ophthalmology, First Affiliated Hospital of Jinan University, Guangzhou, China; 3 Department of Ophthalmology, Sixth Affiliated Hospital of Jinan University, Dongguan, China; 4 Institute of Vision Research, Department of Ophthalmology, Yonsei University College of Medicine, Seoul, South Korea

**Keywords:** Cell biology, Environmental sciences, Biomarkers, Medical research

## Abstract

The impact of particulate matter (PM) on ocular surface health has attracted increased attention in recent years. Previous studies have reported that differences in the chemical composition of PM can affect the toxicological response. However, available information on the toxic effects of chemical components of PM on the ocular surface is insufficient. In this paper, we aimed to investigate the toxicity effects of chemical components of PM on the ocular surface, focusing on the effects of four different types of nanoparticles (NPs) in human corneal epithelial cells (HCECs) and human conjunctival epithelial cells (HCjECs), which include titanium dioxide (TiO_2_), carbon black (CB), zinc dioxide (ZnO), and silicon dioxide (SiO_2_). We found that the in vitro cytotoxic effects of CB, ZnO, and SiO_2_ NPs are dependent on particle properties and cell type as well as the exposure concentration and time. Here, the order of increasing toxicity was SiO_2_ → CB → ZnO, while TiO_2_ demonstrated no toxicity. Moreover, toxic effects appearing more severe in HCECs than HCjECs. Reactive oxygen species (ROS)-mediated oxidative stress plays a key role in the toxicity of these three NPs in HCECs and HCjECs, leading to apoptosis and mitochondrial damage, which are also important contributors to aging. Sirtuin1 (SIRT1) as an NAD+-dependent protein deacetylase that seems to play a potential protective role in this process. These findings implied that ROS and/or SIRT1 may become a potential target of clinical treatment of PM- or NP-related ocular surface diseases.

## Introduction

Particulate matter (PM) is an important component of air pollution that affects ocular health^1^. A growing number of studies have shown that exposure to elevated PM concentrations is linked with an increased frequency of outpatient visits for ocular surface diseases, including nonspecific conjunctivitis, blepharitis, pterygium, and dry eye disease^2–5^. The chemical components of PM include mineral matter [e.g., oxides of aluminum (Al), calcium (Ca), titanium, silicon, zinc, iron (Fe), and magnesium (Mg), etc.], organic matter, elemental carbon, secondary inorganic aerosol, sea salt, and trace elements^6^. Previous studies have reported that the complex and variable composition of PM can lead to differences in health effects^7^. In particular, heavy metals are considered to contribute significantly to PM-related cytotoxicity^8^. However, few studies to date have focused on the different effects of these chemical components of PM on eyes.

A number of different types of nanoparticles (NPs), including titanium dioxide (TiO_2_) NPs, carbon black (CB) NPs, and silicon dioxide (SiO_2_) NPs, which can mimic different chemical components of PM, are often adopted in studies seeking to investigate the health effects of PM^9–14^. Oxidative stress plays an important role in the toxic effects of PM or NPs and has been extensively studied both in vitro and in vivo^15–20^. Previous studies have shown that both PM or NPs can increase the production of reactive oxygen species (ROS) and apoptosis in in vivo cornea cells^19,21,22^. In addition, Gao et al. reported that PM can induce DNA damage and cell senescence in human corneal epithelial cells (HCECs)^23^. It is well known that DNA damage, cellular senescence, and mitochondrial dysfunction as well as the production of ROS or inflammation are hallmarks of aging^24^. Recently, studies have shown that PM or SiO_2_ NPs can damage human pulmonary fibroblast cells or bronchial epithelial cells through attenuates the expression of sirtuin1 (SIRT1)^25,26^. SIRT1 is an NAD+-dependent protein deacetylase that has been linked to various physiological and pathological processes and conditions, including DNA repair, aging, cellular senescence, oxidative stress, inflammation, and mitochondrial function^27–31^. Therefore, in the field of aging mechanisms and intervention, SIRT1 has been a topic of interest in recent years. However, the role of SIRT1 in ocular surface injury induced by PM or NPs remains unclear.

To evaluate the different effects of chemical components of PM on the ocular surface, this study conducted a systematic and comparative investigation of the toxicity effects of four different NPs (TiO_2_, CB, ZnO, and SiO_2_) on HCECs and human conjunctival epithelial cells (HCjECs). The toxic effects of these NPs on HCECs and HCjECs were investigated. In addition, the expression of SIRT1 as well as ROS generation, apoptosis, and mitochondrial function were investigated.

## Materials and methods

### Reagents

Keratinocyte-serum free medium (K-SFM), keratinocyte supplements containing human recombinant epidermal growth factor (EGF) and bovine pituitary extract, phenol red-free Dulbecco's modified Eagle medium, 0.05% Trypsin-ethylenediaminetetraacetic acid, fetal bovine serum, 1 × phosphate-buffered saline (PBS), and penicillin–streptomycin (10,000 U/mL) were purchased from Thermo Fisher Scientific (Waltham, MA, USA). Roswell Park Memorial Institute (RPMI)-1640 medium and bovine serum albumin (BSA) were obtained from Sigma-Aldrich Corp. (St. Louis, MO, USA). Fibronectin was purchased from Corning (Corning, NY, USA). Rat tail collagen type I was obtained from Advanced BioMatrix (San Diego, CA, USA). Water-soluble tetrazolium salt (WST)-8 cell viability kit was purchased from DoGen Bio (Seoul, Korea). The ApopTag Red in situ apoptosis detection kit was obtained from Millipore Sigma (Burlington, MA, USA). The tetramethylrhodamine ethyl ester (TMRE) mitochondrial membrane potential (MMP) assay kit (#ab113852) and 2′,7′-dichlorofluorescein diacetate (DCFH-DA) cellular ROS detection assay kit (#ab113851) were purchased from Abcam (Cambridge, England). Radioimmunoprecipitation assay buffer (#89901) was obtained from Thermo Fisher Scientific. Anti-SIRT1 (#8469) and β-actin (#5125) antibodies were purchased from Cell Signaling Technology (Danvers, MA, USA). Anti-mouse immunoglobulin G horseradish peroxidase–linked antibody (#PI-2000) was obtained from Vector Laboratories (Burlingham, NY, USA). Finally, eight-well black cell culture slides, 96-well black plates, and six-well and 96-well cell culture plates were purchased from SPL Life Sciences (Pocheon, Korea).

### Particle preparation

Manufactured TiO_2_, CB, ZnO, and SiO_2_ NPs were purchased from the commercial suppliers indicated in Table 1. All particle samples were weighted on a high precision microbalance and a stock suspension was performed in sterile PBS at a concentration of 10 mg/mL. Prior to dilution in cell culture media, these suspensions were sonicated four times intermittently (for five minutes every 10 min) through a bath sonicator, then additionally sonicated three times for 20 s within 5 min prior to their experimental use so as to minimize their aggregation.

**Table 1:** Parameters of the four NPs.

Particle	Supplier	Material code	Size
TiO_2_	Sigma-Aldrich Corp. (St. Louis, MO, USA)	#634662	< 100 nm
CB	Orion Engineered Carbons (Piscataway, NJ, USA)	Printex55	25 nm
ZnO	Sigma-Aldrich Corp. (St. Louis, MO, USA)	#544906	< 100 nm
SiO_2_	Sigma-Aldrich Corp. (St. Louis, MO, USA)	#637238	10–20 nm

### Cell culture and treatment

HCECs (2.040 pRSV-T, CRL-11516™) and HCjECs (clone 1-5c-4 [Wong-Kilbourne derivative (D) of Chang conjunctiva], KCLB No. 30052) were obtained from the American Type Culture Collection (ATCC; Manassas, VA, USA) and Korean Cell Line Bank (KCLB, Seoul, Korea), respectively. The HCECs were maintained in K-SFM supplemented with 0.05 mg/mL of bovine pituitary extract, 5 ng/mL of EGF, 100 U/mL of penicillin, and 100 μg/mL of streptomycin at 37 °C in a 5% CO_2_ incubator. The flasks used were precoated overnight in a 37 °C incubator, using a mixture of 0.01 mg/mL of fibronectin, 0.03 mg/mL of bovine collagen type I, 0.01 mg/mL of BSA), and RPMI-1640. Before this step, the coating solution was discarded, and the flasks were kept in a biosafety hood for 15 min at room temperature. The HCjECs were maintained in RPMI-1640 containing 10% fetal bovine serum, 100 U/mL of penicillin, and 100 μg/mL of streptomycin at 37 °C in a 5% CO_2_ incubator.

For experiments, HCECs (2 × 10^5^ cells/mL) and HCjECs (2 × 10^5^ cells/mL) were seeded in 96-well, 24-well, and six-well plates with 100 µL, 500 µL, and 2500 µL of culture medium, respectively. Cells were incubated for 24 h to reach 90% confluency and treated with NPs at the indicated concentration. The cellular responses were examined after first exposure and the results compared with those of untreated cells (control group).

### Cytotoxicity tests

Cytotoxicity was evaluated using a WST-8 cell viability assay kit. The cells were incubated with or without different concentrations of each NP (12.5, 25, 50, 100, 200, or 400 μg/mL) for 24 h. The WST-8 solution (DoGen Bio, Seoul, Korea) was added to each well and cells were incubated for two hours; then, the colored supernatants were measured at 450 nm. The results are presented as percentages relative to the untreated control. All experiments were performed in triplicate.

In addition, to evaluate cytotoxicity with different exposure times, cells were incubated with each NP for 1, 3, 6, and 24 h with a relatively high concentration of 100 μg/mL. The half-maximal inhibitory concentration 50 (IC_50_), or the concentration of NPs at which 50% of cells were nonviable, was determined. Data are presented as average IC_50_ values for each test NP, respectively.

### Cellular uptake or interaction with NPs

The degree of NPs uptake or adsorption on cellular membranes was examined by analyzing forward scatter (FSC) verses side scatter (SSC) using flow cytometry (LSRFortessa X-20 program (BD Biosciences, San Jose, CA, USA) as described previously^32^. Briefly, cells were treated with 100 μg/mL of each NPs for 6 h, and then trypsinized and suspended in PBS for flow cytometry. Following gating, control and NPs-exposed cells were run and plotted to examine increase in side scatter (SSC) due to endocytotic or adsorptive NPs interaction.

### Intracellular generation of ROS

DCFH-DA was used to measure intracellular ROS using the DCFH-DA cellular ROS detection assay kit according to the manufacturer's protocol. Cells were treated with 100 μg/mL of each NP for 3 h. Diluted DCFH-DA (10 μM) was then added to the cells, which were subsequently incubated for 30 min in the dark. A total of 10,000 events (cells) were analyzed by flow cytometry using the LSRFortessa X-20 program (BD Biosciences, San Jose, CA, USA) at excitation/emission wavelengths of 488/535 nm. The results were expressed as percentages of increase over control cells (as 100%).

### Cell apoptosis analysis

Cells were seeded on cover slips in eight-well black cell culture slides and cultured overnight to allow for attachment. After exposure to 100 μg/mL of each NP for 6 h, the apoptotic cell count was detected using the TUNEL kit (ApopTag Red in situ apoptosis detection kit) according to the manufacturer's instructions. The cell nuclei were stained with 4′,6-diamidino-2-phenylindole. Images were captured with the LSM 700 laser scanning confocal microscope (Carl Zeiss, Jena, Germany). The percentage of TUNEL-positive cells was calculated in the total cells of each digital image (scale bar = 50 µm) of each group.

### Detection of MMP

Cells were seeded in 96-well black plates and cultured overnight to allow for attachment. After exposure to 100 μg/mL of each NP for 6 h, the MMP was assessed using the TMRE assay. Cells were incubated for 30 min with TMRE at 500 nM diluted in warm culture medium at 37 °C and 5% CO_2_. Cells were then washed twice with warm PBS/0.2% BSA and fluorescence intensity was detected using a plate reader with excitation/emission wavelengths at 549/575 nm.

### Protein extraction and western blotting

Cells were seeded in six-well plates and cultured overnight to allow for attachment. After exposure to 100 μg/mL of each NP for 6 h, cells were lysed in radioimmunoprecipitation assay buffer for 30 min on ice. Protein concentrations were determined using the bicinchoninic acid method. Proteins were separated by 8% polyacrylamide gel electrophoresis containing 0.1% sodium dodecyl sulfate and transferred to polyvinylidene difluoride membranes. The nitrocellulose membrane was blocked with 5% BSA and subsequently incubated with SIRT1 monoclonal antibody (1:1000 dilution) overnight at 4 °C. Then, the membranes were incubated with horseradish peroxidase-conjugated horse anti-mouse immunoglobulin G secondary antibody (1:2000 dilution) for another hour. β-actin (1:10,000 dilution) was used as the loading control, and the ImageJ software (National Institutes of Health, Bethesda, MD, USA) was used to calculate protein intensity. The results of Western blot analysis were expressed as a ratio to β-actin, as previously described^10–13^.

### Statistical analyses

All data were expressed as the mean ± standard deviation (SD) values of at least three independent experiments. Statistical analyses were performed using one-way analysis of variance with post-hoc Tukey’s test using the Statistical Package for Social Sciences version 20.0 program (IBM Corp., Armonk, NY, USA). Results were considered statistically significant at a p-value of less than 0.05.

## Results

### Cytotoxicity induced by NPs

**Figure 1:**
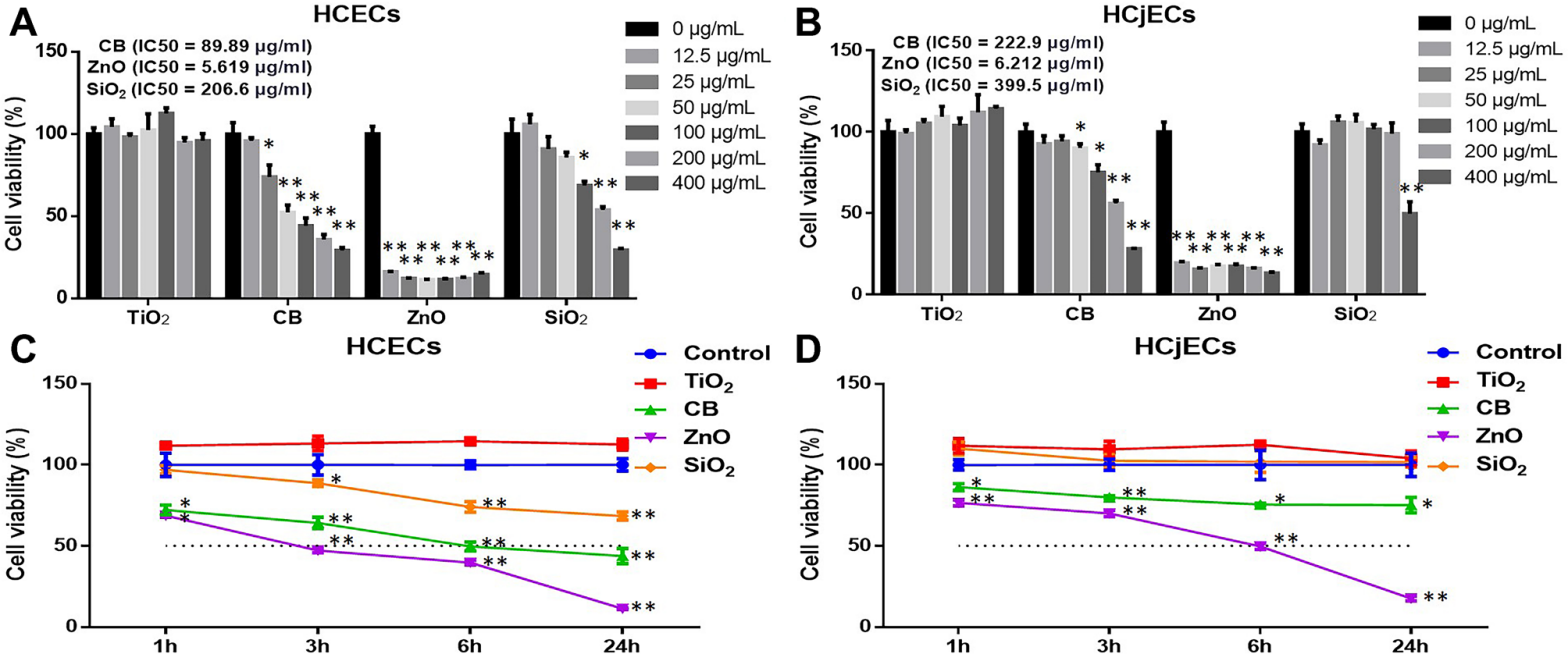
Cytotoxicity evaluation of TiO_2_, CB, ZnO, and SiO_2_ NPs with the WST-8 assay. HCECs (**A**) and HCjECs (**B**) were treated with different concentrations (12.5, 25, 50, 100, 200, or 400 μg/mL) of each NP for 24 h. The IC_50_ values of each NP are shown at the top left of A and B. In addition, HCECs (**C**) and HCjECs (**D**) were treated with 100 μg/mL of each NP for different lengths of time (1, 3, 6, and 24 h) and their viability was measured with the WST-8 assay. Results are given in percentages related to untreated control cells. Results are shown as the mean ± standard deviation values of three independent experiments, each of which was carried out in triplicate. *p < 0.05 and **p < 0.001 vs. untreated controls.

We studied and compared the toxicity effects of four NPs in HCECs and HCjECs two cell lines. As expected, we found that cell toxicity is dependent on exposure concentration, particle properties, and cell type. The order of increasing toxicity of the tested NPs was SiO_2_ → CB → ZnO. After 24 h of exposure at varying doses (12.5, 25, 50, 100, 200, and 400 μg/mL) of TiO_2_, CB, ZnO, and SiO_2_ NPs, the cell viability of HCECs and HCjECs as detected by the WST-8 assay resulted in explicit dose-dependent reductions, excluding TiO_2_ NPs (Fig. 1A,B). Especially, ZnO NPs were shown to impart significant (p < 0.001) greater toxicity than the control treatment on both HCECs and HCjECs, even at the lowest dose (12.5 μg/mL), with IC_50_ values of 5.169 and 6.212 μg/mL, respectively (Fig. 1A,B). Conversely, toxicity with TiO_2_ NPs was not observed in either cell line even with at the highest dose (400 μg/mL) (Fig. 1A,B). Meanwhile, HCECs were shown to be more susceptible to CB NPs than HCjECs in that the concentration of CB NPs able to cause toxicity in HCECs (25 μg/mL) was lower than that for HCjECs (50 μg/mL), with IC_50_ values of 89.89 and 222.9 μg/mL, respectively (Fig. 1A,B). The concentration of SiO_2_ NPs causing toxicity in HCECs (100 μg/mL) was also lower than that for HCjECs (400 μg/mL), with IC_50_ values of 206.6 and 399.5 μg/mL, respectively (Fig. 1A,B).

In addition, we investigated the toxicity effects of NPs at the concentration of 100 μg/mL at different time points (1, 3, 6 and 24 h). The viability of HCECs and HCjECs resulted in an explicit time-dependent reduction, excluding TiO_2_ NPs (Fig. 1C,D). The toxicity of CB and ZnO NPs on HCECs and HCjECs could be observed as early as 1 h after treatment (Fig. 1C,D). However, that of SiO_2_ NPs was only observed in HCECs after 3 h after first exposure, and there were no toxic effects of SiO_2_ NPs on HCjECs, even after 24 h of exposure (Fig. 1C,D). In addition, ZnO NPs lessened cell viability by greater than 50% in HCECs (47.2%) and HCjECs (49.7%) after 3 and 6 h of exposure, respectively (Fig. 1C,D), while CB NPs did so in HCECs (49.7%) 6 h after first exposure, but cell viability in HCjECs remained at 75.2% even 24 h after first exposure to CB NPs (Fig. 1C,D). These results indicate that HCECs are more vulnerable to these NPs than HCjECs.

### NPs uptake in HCECs and HCjECs

**Figure 2:**
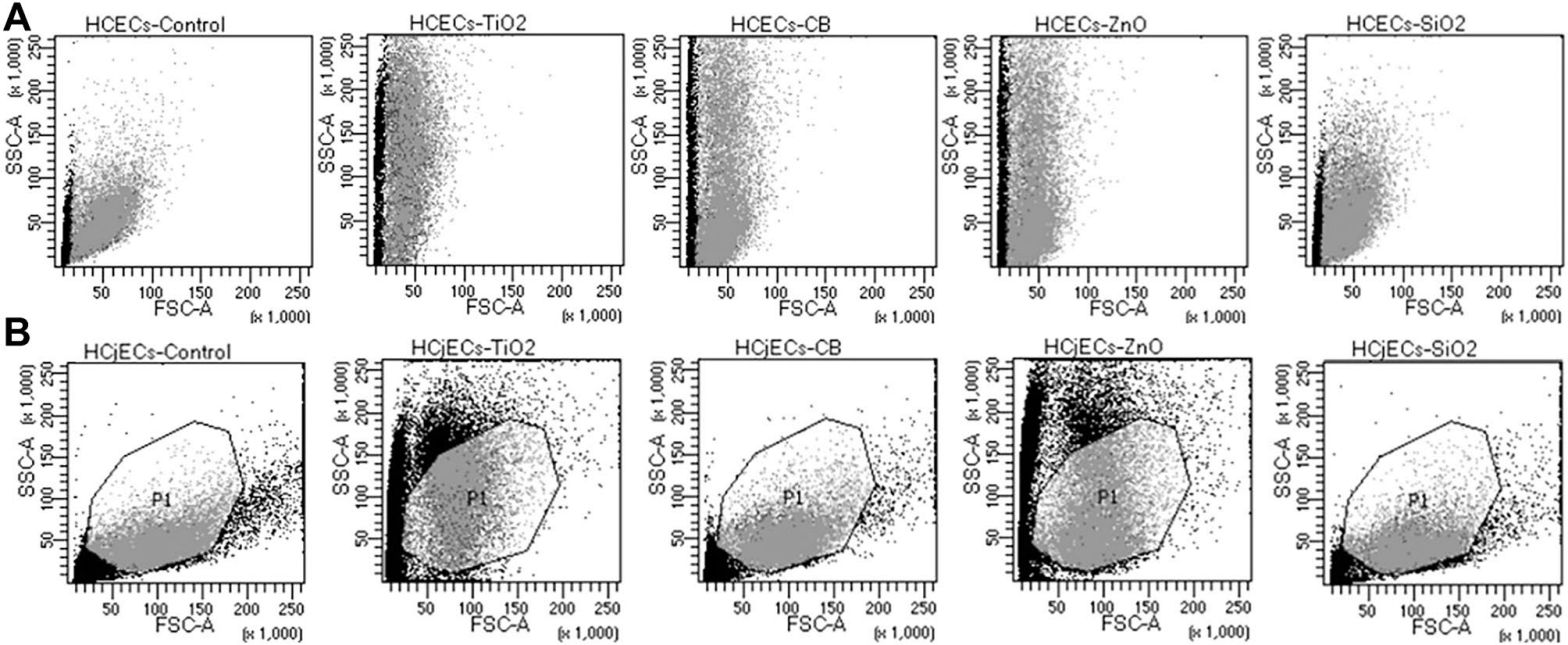
Analysis of NPs uptake was evaluated by analyzing FSC verses SSC using flow cytometry. HCECs (**A**) and HCjECs (**B**) were treated with 100 μg/mL of TiO_2_, CB, ZnO, and SiO_2_ NPs for 6 h. NPs uptake/surface adsorption is reflected by increases in the side scatter of cell populations (relative to controls).

Uptake or attachment of NPs to cellular membranes in HCECs and HCjECs was examined by analyzing FSC verses SSC using flow cytometry. After 6 h of exposure to 100 μg/mL of the four NPs, we see that NPs had a variable effect on the side scatter of the cells, with all two cell types exhibiting substantial uptake or interaction with TiO_2_ and ZnO NPs as evident by a large increase in side scatter/left shift (nanoparticle uptake-associate cell size reduction) (Fig. 2). In addition, CB NPs triggered substantial increase in side scatter compared to controls only in HCECs, but not in HCjECs (slight left shift) (Fig. 2). On the other hand, SiO_2_ NPs triggered slight increase in side scatter compared to controls in both HCECs and HCjECs (Fig. 2).

### Intracellular ROS generation induced by NPs

**Figure 3:**
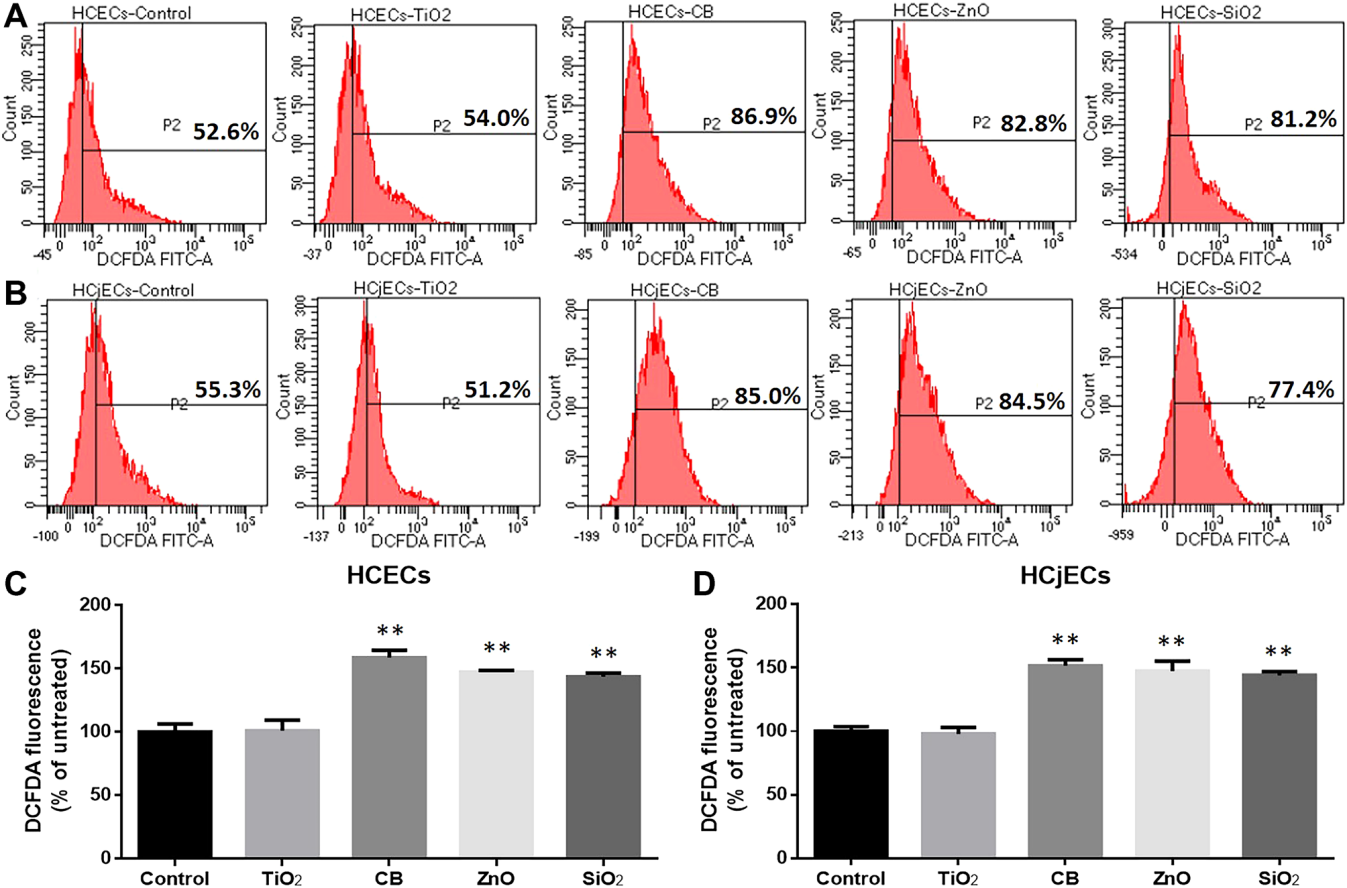
Intracellular ROS generation of cells was evaluated by DCFDH-DA oxidation assay. HCECs (**A**, **C**) and HCjECs (**B**, **D**) were treated with 100 μg/mL of TiO_2_, CB, ZnO, and SiO_2_ NPs for 3 h. The representative figures are shown, and DCFDA-positive cells (P2) were calculated as a percentage of the controls. Results are shown as the mean ± standard deviation values of three independent experiments, each of which was carried out in triplicate. *p < 0.05 and **p < 0.001 vs. untreated controls.

ROS has been suggested to be an important biomarker in the evaluation of nanotoxicity^33^. We measured the production of intracellular ROS via DCFDH-DA oxidation assay, which is used to quantitatively assess ROS in live cell samples. Thus, we measured the ROS generation at an earlier time point (3 h) following exposure to NP (100 μg/mL). At this point, CB, ZnO, and SiO_2_ NPs significantly increased the DCF fluorescence intensity relative to the control treatment in both HCECs and HCjECs. However, there were no significant changes in cells exposed to TiO_2_ NPs (Fig. 3).

### Apoptosis induced by NPs

**Figure 4:**
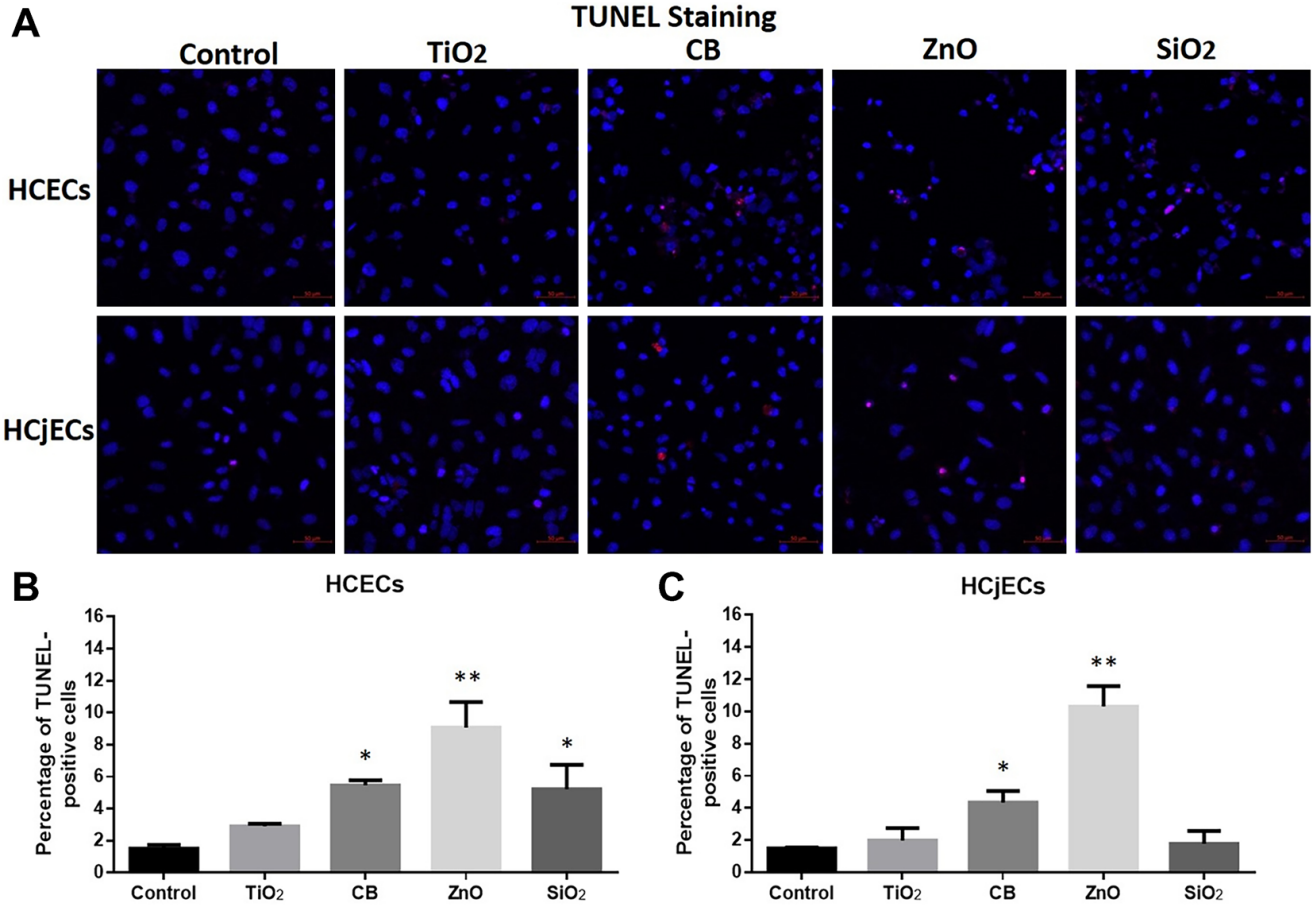
Cellular apoptosis was detected by TUNEL staining. (**A**) Representative TUNEL fluorescence images of HCECs and HCjECs treated with 100 μg/mL of TiO_2_, CB, ZnO, and SiO_2_ NPs for 6 h. (**B**) Percentage of TUNEL-positive cells in HCECs. (**C**) Percentage of TUNEL-positive cells in HCjECs. Cell nuclei are labeled with DAPI (blue) and TUNEL positive cells fluoresced red. Results are shown as the mean ± standard deviation values of three independent experiments, each of which was carried out in triplicate. *p < 0.05 and **p < 0.001 vs. untreated controls.

Detection and quantification of apoptosis were carried out by TUNEL assay. Six hours after first exposure to 100 μg/mL of the four NPs, the percentage of TUNEL-positive cells were significantly (p < 0.05) increased in CB-exposed and ZnO-exposed cells relative to with control treatment among both HCECs and HCjECs (Fig. 4). However, SiO_2_ NPs significantly (p < 0.05) increased apoptosis only among HCECs, not HCjECs (Fig. 4).

### Changes in MMP induced by NPs

**Figure 5:**
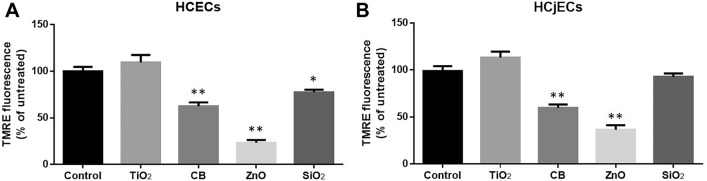
Mitochondrial membrane potential was measured by using the TMRE assay. HCECs (**A**) and HCjECs (**B**) were treated with 100 μg/mL of TiO_2_, CB, ZnO, and SiO_2_ NPs for 6 h. The results were calculated as a percentage of TMRE fluorescence intensity relative to controls. Results are shown as the mean ± standard deviation values of three independent experiments, each of which was carried out in triplicate. *p < 0.05 and **p < 0.001 vs. untreated controls.

MMP was assessed using the TMRE assay. After 6 h of exposure to 100 μg/mL of the four NPs, TMRE fluorescence intensity was significantly decreased in CB-exposed and ZnO-exposed cells relative to among control cells in both HCECs and HCjECs (p < 0.001) (Fig. 5). However, SiO_2_ NPs significantly reduced the TMRE fluorescence intensity only among HCECs (p < 0.05), not HCjECs (Fig. 5).

### Changes in SIRT1 expression induced by NPs

**Figure 6:**
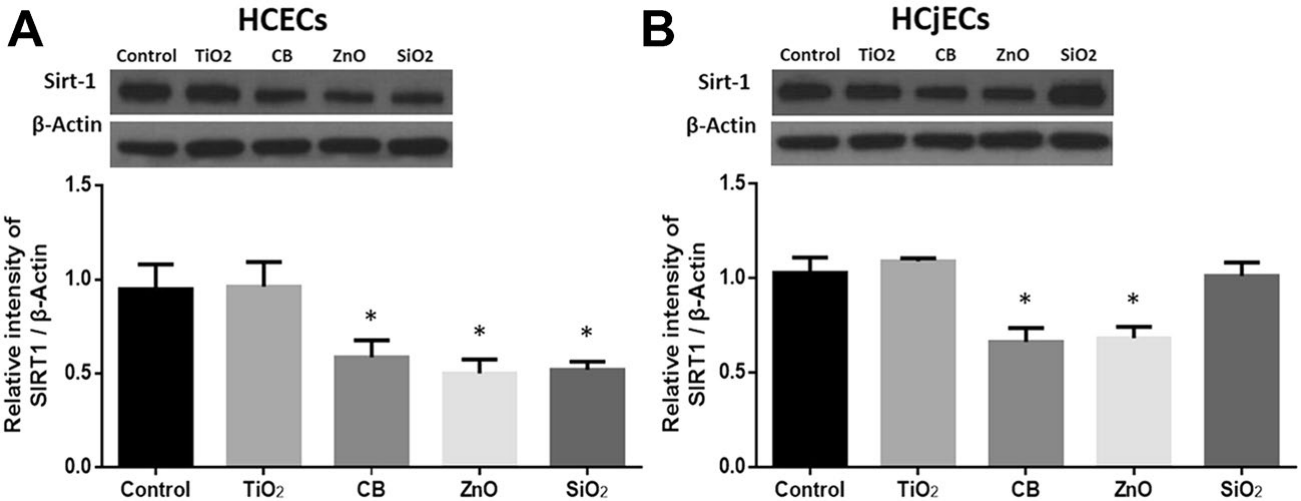
Expression of SIRT1 was evaluated by western blot analyses. HCECs (**A**) and HCjECs (**B**) were treated with 100 μg/mL of TiO_2_, CB, ZnO, and SiO_2_ NPs for 6 h. A representative example of a western blot gel image is shown at the top left of each figure (full length blot is presented in Supplementary Fig. 2). β-actin was used as an internal loading control. Results are expressed as a ratio to β-actin concentration and the mean ± standard deviation values of three independent experiments, each of which was carried out in triplicate. *p < 0.05 and **p < 0.001 vs. untreated controls.

SIRT1 expression was measured by western blotting in HCECs and HCjECs after exposure to 100 μg/mL of four NPs for 6 h. After NP exposure, SIRT1 expression was significantly decreased in CB-exposed and ZnO-exposed cells relative to after control treatment in both HCECs and HCjECs (p < 0.05) (Fig. 6). However, SiO_2_ NPs significantly (p < 0.05) reduced SIRT1 expression only among HCECs, not HCjECs (Fig. 6).

## Discussion

In recent years, the impact of PM on ocular surface health has attracted increased attention^1,34^. Previous studies have reported that differences in the chemical composition of PM can affect the toxicological response^35^. For example, Yuan et al. reported that heavy metals components such as Zn, chromium (Cr), manganese (Mn), Fe, copper (Cu), and lead (Pb) are considered to make a significant contribution to PM-related cytotoxicity in human lung epithelial cells^8^. However, available information on the toxic effects of chemical components of PM on the ocular surface is insufficient. NPs as surrogate particles for PM are widely used to explore the biological effects of PM. Previous studies have indicated that the cytotoxicity of NPs depends upon various factors such as cell type, particle properties, and exposure concentration and time^18,32,36^. Recently, Lee et al. evaluated the toxicity of five different types [silver (Ag), cerium dioxide (CeO_2_), SiO_2_, TiO_2_, and ZnO] of NPs on rabbit cornea cells at different doses (1, 10, 12.5, 25, 50, and 100 μg/mL) for 24 hours^21^. These authors found that only Ag and ZnO NPs reduced cell viability following exposure at the highest concentration (100 μg/mL), while the other tested NPs had no toxicity effect on rabbit cornea cells^21^. However, the toxicity effects of TiO_2_ and SiO_2_NPs (treatment of 100 μg/mL for 24 h) have been shown by other in vitro studies, which evaluated such in mouse corneal endothelial cells and HCECs, respectively^14,22^. Thus, the cytotoxicity of NPs in the eye seems to highly depend on the cell type being considered.

This study evaluated the toxicity effect of four different NPs (TiO_2_, CB, ZnO, and SiO_2_) on cultured HCECs and HCjECs by cell viability assay and found that CB, ZnO, and SiO_2_ NPs significantly reduced cell viability in a dose-dependent manner in both HCECs and HCjECs after 24 h exposure at varying doses of each NP. As shown by our results, the order of increasing toxicity of the tested NPs was SiO_2_ → CB → ZnO. In addition, this study found that toxicity of CB and SiO_2_ NPs on HCECs seems to be greater than that on HCjECs given that the IC_50_ value of these two NPs on HCjECs was twice as high as that on HCECs. However, the toxicity of ZnO NPs seems similar in two cell lines and is the greatest level of toxicity recorded in this study. Moreover, we evaluated the cytotoxicity of NPs at four different exposure times (1, 3, 6, and 24 h) using the concentration of 100 μg/mL and found that the toxicity induced by these NPs is also dependent on exposure time. Interestingly, 100 μg/mL of SiO_2_ NPs was only toxic in HCECs, and the extent to which CB and ZnO NPs reduce cell viability over time was more obvious in HCECs than in HCjECs. Therefore, these results indicate that the toxic effects of NPs on both HCECs and HCjECs depend upon the particle properties and exposure concentration and time, which are consistent with the findings of a previous study involving rabbit cornea cells^21^. In addition, we found that the toxic effects of CB and SiO_2_ NPs are highly dependent on the cell type in this study. However, if other NPs exhibit a high degree of toxicity like that of ZnO, the dependence of such on cell type seems to be weakened.

The generation of ROS and the subsequent production of oxidative stress is a predominant mechanism leading to nanotoxicity, including DNA damage, genotoxicity, apoptosis, and carcinogenesis^16,33,37,38^. In addition, mitochondria are the main site of cellular ROS production, and any structural and functional disorders can lead to mitochondrial ROS accumulation, which is the main inducer of apoptosis^39,40^. Therefore, to explore the role of oxidative stress in NP-induced cytotoxicity, this study evaluated ROS production, cell apoptosis, and mitochondrial membrane potential by using DCFDH-DA assay, TUNEL staining, and TMRE assay, respectively. On the other hand, ROS generation has been suggested to be an initial cellular response to NP internalization and, later, cell death^41^. Based on our toxicity test results, this study evaluated ROS as well as apoptosis and mitochondrial damage after 3 and 6 h of exposure, respectively. This study found that all positive NPs in the toxicity test induced ROS generation as well as apoptosis and mitochondrial damage when exposed to a dose of 100 μg/mL for 6 h. However, we found that SiO_2_ NPs did not induce any cellular response in HCjECs, although there was an increase in ROS production. ROS play a dual role in the fate of cells, i.e., causing cell death as well as acting as second messengers to induce an adaptive cell response^42^. Interestingly, a recent study showed that SiO_2_ NPs induce a slight increase of intracellular ROS but can activate mTOR and autophagy that avoids possible cellular damage induced by SiO_2_ NPs^43^. Therefore, these results indicate that oxidative stress plays a key role in the properties and cell type–dependent toxicity of NPs, which is closely related to the production of ROS as well as potential defense mechanisms.

SIRT1, a member of the sirtuin family, is a highly conserved NAD + ‑dependent histone deacetylase and plays a crucial role in cell survival under oxidative stress conditions through directly regulating multiple targets, including eNOS, peroxisome proliferator-activated receptor-γ coactivator 1-α (PGC1α), p53, Forkhead Box O family (FoxO), and NF-Κb^44–49^. On the contrary, excessive ROS and/or oxidative stress conditions can inhibit the expression of SIRT1, thereby promoting cell apoptosis^30,50,51^. Previous studies have reported that exposure to NPs can inhibit the expression of SIRT1 to promote cell apoptosis, mitochondrial damage, and inflammation^26,52^. However, these injuries can be reversed by upregulating or activating SIRT1^26,52^. In this study, we found that all positive NPs in the toxicity test could reduce the expression of SIRT1 following exposure to a dose of 100 μg/mL for 6 h. However, we also found that SiO_2_ did not reduce the expression of SIRT1 in HCjECs, although ROS production increased. Therefore, these results indicate that the expression of SIRT1 plays a potential protective role in the process of NP-induced, ROS-mediated oxidative stress, but excessive ROS can still inhibit the expression of SIRT1 to promote cell apoptosis and mitochondrial damage. It would be interesting to test whether the upregulation or activation of SIRT1 could reduce NP-induced oxidative stress in HCECs and HCjECs. Further studies are required to understand the protective mechanisms of SIRT1 on nanotoxicity in the eyes.

In conclusion, the results of the current study confirmed that the in vitro cytotoxic effects of CB, ZnO, and SiO_2_ NPs are dependent on particle properties and cell type as well as the exposure concentration and time. In HCECs and HCjECs, ZnO NPs possessed the greatest toxicity, while TiO_2_ NPs demonstrated no toxicity. We also showed that CB and SiO_2_ NPs have different cytotoxic potencies in HCECs and HCjECs, while that of ZnO NPs was similar between the two cell types. HCECs seem to be more vulnerable to these NPs (as PM surrogate particles) than HCjECs. ROS-mediated oxidative stress plays a key role in the toxicity of NPs in HCECs and HCjECs, leading to apoptosis and mitochondrial damage. On the other hand, this study speculate that these NPs may promote aging effect to HCECs and HCjECs, as supported by the elevation of ROS production and mitochondrial damage as well as reduction of SIRT1 expression, which are indirect evidence to aging. SIRT1 seems to play a potential protective role in toxicity and aging-related effects of NPs to HCECs and HCjECs. In summary, our study provides evidence of a potential mechanism that may induce toxic and aging-related effects on the ocular surface induced by different chemical components of PM, through the use of four different types of NPs. These findings may provide valuable insights regarding the treatment of PM- or NP-related ocular surface injury in the future.

## Supplementary Information

Supplementary Information.
