# Supplementary material for: Comparison of cytotoxicity effects induced by four different types of nanoparticles in human corneal and conjunctival epithelial cells
Source: Sci Rep. 2022 Jan 7;12:155. doi: 10.1038/s41598-021-04199-3 (PMC8742118; doi:10.1038/s41598-021-04199-3)
Supplement: Supplementary file 1 — Supplementary Information. [file 41598_2021_4199_MOESM1_ESM.docx]

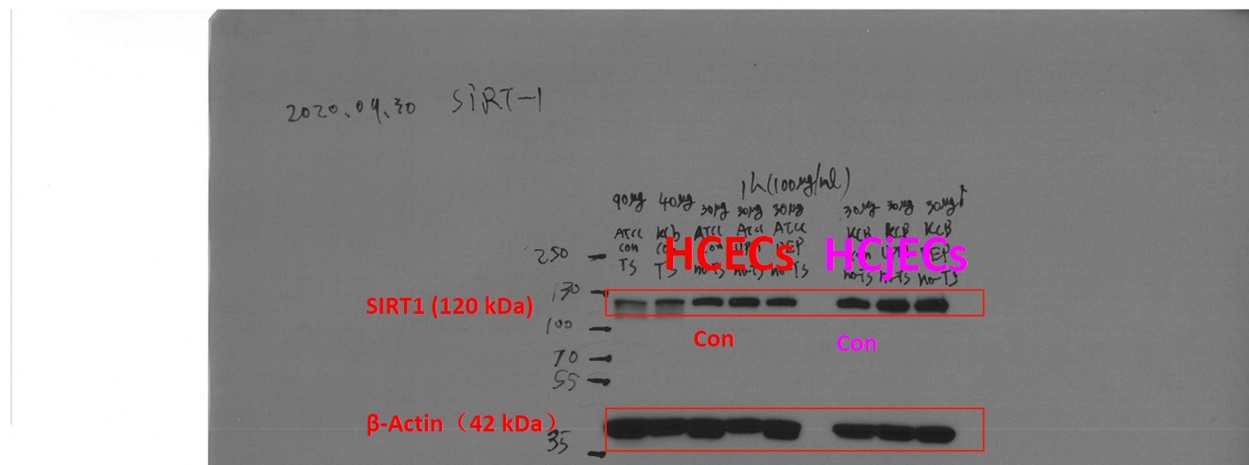


**Supplementary Fig.1 Validation of SIRT1 protein levels in human corneal epithelial cells (HCECs) and human conjunctival epithelial cells (HCjECs) in our preliminary experiment.** β-actin and SIRT1 shown their bands of western Blot analysis around 42kDa and 120kDa, respectively.


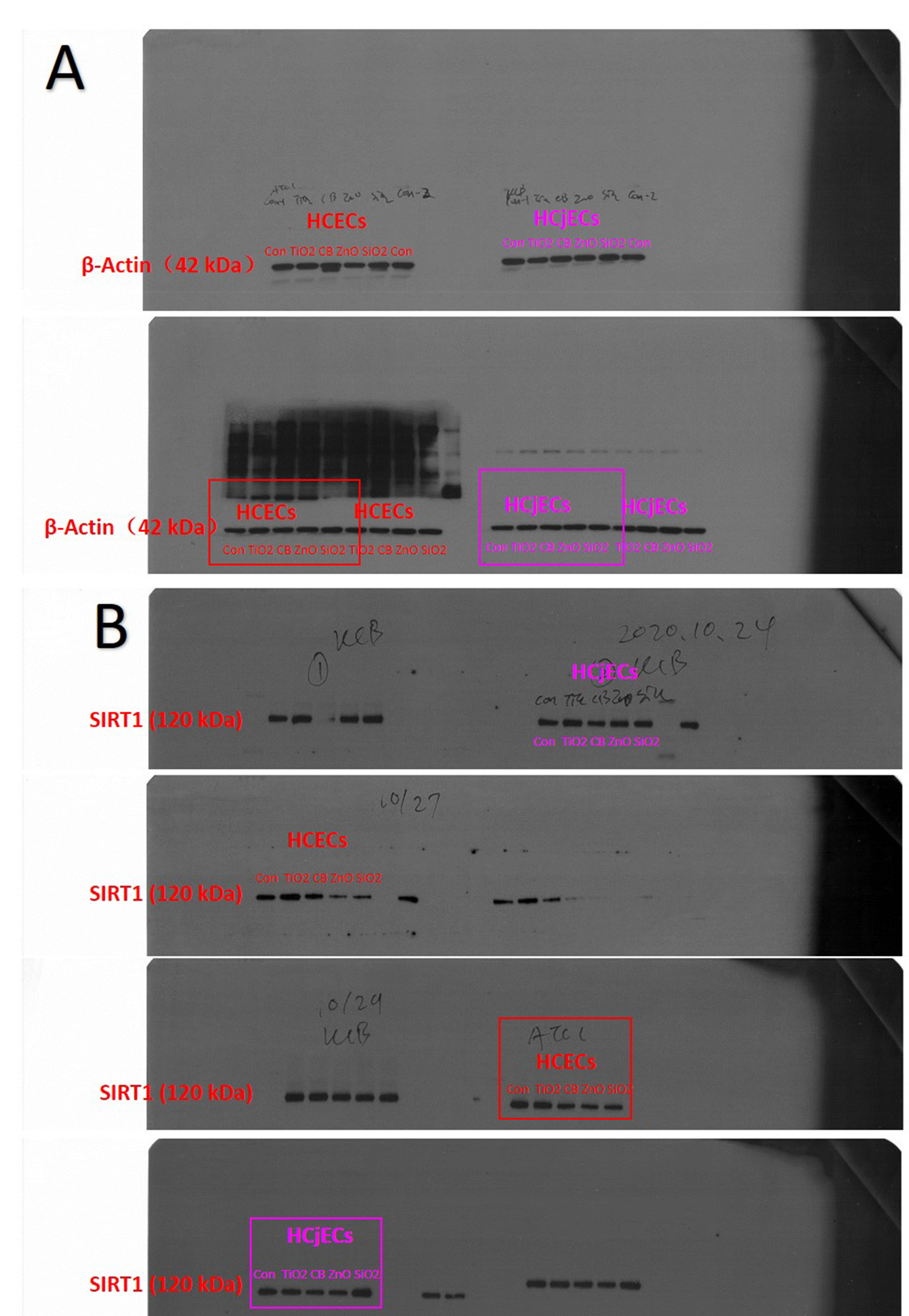


**Supplementary Fig.2 Validation of SIRT1 protein levels in HCECs and HCjECs following 6 hours of NPs exposure.** Protein lysates (30μg) of cells were applied to SDS-PAGE. After gel electrophoresis and protein transferring, we cut the membrane as two parts according to molecular size. β-actin (1:10,000 dilution) (A) and SIRT1 monoclonal antibody (1:1,000 dilution) (B) were incubated. β-actin served as a loading control.
